# Selection signature analysis and genome-wide divergence of South African Merino breeds from their founders

**DOI:** 10.3389/fgene.2022.932272

**Published:** 2023-01-04

**Authors:** E. F. Dzomba, M. A. Van Der Nest, J. N. T. Mthembu, P Soma, M. A. Snyman, M. Chimonyo, F. C. Muchadeyi

**Affiliations:** ^1^ Discipline of Genetics, School of Life Sciences, University of KwaZulu-Natal, Durban, South Africa; ^2^ Agricultural Research Council Biotechnology Platform, Private Bag X5 Onderstepoort, Pretoria, South Africa; ^3^ Agricultural Research Council, Animal Production and Improvement, Pretoria, South Africa; ^4^ Grootfontein Agricultural Development Institute, Middelburg, South Africa; ^5^ Discipline of Animal and Poultry Science, School of Agricultural, Earth and Environmental Sciences, University of KwaZulu-Natal, Durban, South Africa

**Keywords:** Merino-type sheep, population genetic structure, breed divergence, SNP genotypes, selection sweeps, EHH signatures

## Abstract

Merino sheep are a breed of choice across the world, popularly kept for their wool and mutton value. They are often reared as a pure breed or used in crossbreeding and are a common component in synthetic breed development. This study evaluated genetic diversity, population structure, and breed divergence in 279 animals of Merino and Merino-based sheep breeds in South Africa using the Illumina Ovine SNP 50K BeadChip. The sheep breeds analysed included the three Merino-derived breeds of Dohne Merino (*n* = 50); Meatmaster (*n* = 47); and Afrino (*n* = 52) and five presumed ancestral populations of Merinos (Merino (*n* = 46); South African Merino (*n* = 10); and South African Mutton Merino (*n* = 8)); and the non-Merino founding breeds of Damara (*n* = 20); Ronderib Afrikaner (*n* = 17); and Nguni (*n* = 29). Highest genetic diversity values were observed in the Dohne Merino (DM), with *H*_*o*_ = 0.39 ± 0.01, followed by the Meatmaster and South African Merino (SAM), with *H*_*o*_ = 0.37 ± 0.03. The level of inbreeding ranged from 0.0 ± 0.02 (DM) to 0.27 ± 0.05 (Nguni). Analysis of molecular variance (AMOVA) showed high within-population variance (>80%) across all population categories. The first principal component (PC1) separated the Merino, South African Mutton Merino (SAMM), DM, and Afrino (AFR) from the Meatmaster, Damara, Nguni, and Ronderib Afrikaner (RDA). PC2 aligned each Merino-derived breed with its presumed ancestors and separated the SAMM from the Merino and SAM. The *iHS* analysis yielded selection sweeps across the AFR (12 sweeps), Meatmaster (four sweeps), and DM (29 sweeps). Hair/wool trait genes such as *FGF12*; metabolic genes of *ICA1*, *NXPH1*, *and GPR171*; and immune response genes of *IL22*, *IL26*, *IFNAR1*, and *IL10RB* were reported. Other genes include *HMGA*, which was observed as selection signatures in other populations; *WNT5A*, important in the development of the skeleton and mammary glands; *ANTXR2*, associated with adaptation to variation in climatic conditions; and *BMP2*, which has been reported as strongly selected in both fat-tailed and thin-tailed sheep. The DM vs. SAMM shared all six sweep regions on chromosomes 1, 10, and 11 with AFR vs. SAMM. Genes such as *FGF12* on OAR 1:191.3–194.7 Mb and *MAP2K4* on OAR 11:28.6–31.3 Mb were observed. The selection sweep on chromosome 10 region 28.6–30.3 Mb harbouring the *RXFP2* for polledness was shared between the DM vs. Merino, the Meatmaster vs. Merino, and the Meatmaster vs. Nguni. The DM vs. Merino and the Meatmaster vs. Merino also shared an Rsb-based selection sweep on chromosome 1 region 268.5–269.9 Mb associated with the *Calpain* gene, *CAPN7.* The study demonstrated some genetic similarities between the Merino and Merino-derived breeds emanating from common founding populations and some divergence driven by breed-specific selection goals. Overall, information regarding the evolution of these composite breeds from their founding population will guide future breed improvement programs and management and conservation efforts.

## Introduction

Sheep serves as an important source of mutton, manure, and wool, playing an important role in the economy of the country (Groeneveld et al., 2010; Department Agriculture Forestry and Fisheries–South Africa, 2015). Sheep is the ideal farm animal for smallholder farmers due to its small body size that makes it an easily disposable source of meat or cash (Hassan et al., 2014). Furthermore, sheep are sold to meet financial obligations, and their ability to survive in harsh weather conditions allows resource-poor farmers to depend on sheep for food and human livelihood (Hassan et al., 2014; Edea et al., 2017). Sheep also fulfil different socio-cultural roles (Wilson, 2011).

The Merino sheep breed is regarded as one of the oldest and most economically influential sheep breeds in the world (Al-Atiyat et al., 2016). The Merino breeds are known for their fine and soft wool (Department Agriculture Forestry and Fisheries–South Africa, 2015). In South Africa, the breed was introduced in the 1780s from Spain (Buduram, 2004; DAD-IS) and has become adapted to South African climatic and environmental conditions. The South African Merino (SAM) is believed to be a composite breed developed from Spanish, Saxony, Rambouillet, American, and Australian sheep breeds (Mason, 1996) that likely evolved to carry different genes that confer adaptation to particular production environments (Peters et al., 2010). Coupled to selection within breed, several Merino-based breeds have been developed for either wool or mutton or as dual-purpose breeds (Hlophe, 2011).

In South Africa, the Merino breed contributed to the development of composite breeds such as the Afrino (AFR), Meatmaster, and Dohne Merino (DM). The DM breed was developed by crossing the German Mutton Merino (commonly known as SAMM and the SAM ewes) as dual-purpose animals for both meat and wool production (Kotze, 1951; Buduram, 2004; Naidoo et al., 2005; Jordan, 2013). DM sheep are amongst the leading wool sheep breeds in South Africa; they are hardy animals that are well-adapted to their local environments (e.g., resistant to parasites, particularly *Heamonchus contortus*) (Dlamini et al., 2019; Synman and Fisher, 2019). The AFR sheep breed originated during the depressed wool market of the late 1960s when farmers began crossbreeding Merino ewes with mutton breeds. These dual-purpose animals reared for both meat and wool production originated from the crossbreeding of Ronderib Afrikaner (RDA), SAM, and SAMM in a targeted ratio of 25:25:50 of the respective contributing breeds that thrive in the harshest conditions (Bezuidenhout, 2012). The Meatmaster is a breed developed to improve the meat qualities of the fat-tailed sheep breeds (Mason, 1996). Literature indicates that the Meatmaster is a composite of many breeds, though it was predominantly developed from SAMM, SAM, Damara, and other indigenous breeds (Mason, 1996; Hlophe, 2011).

In South Africa, the Merino sheep were bred with local breeds in an effort to improve productivity and resilience of the breeds to the harsh local conditions whilst producing optimally (Bezuidenhout, 2012; Dlamini et al., 2019; Synman and Fisher, 2019). With climate change and other production challenges, the rationale of crossbreeding and developing new and composite breeds will prevail, thus requiring an investigation and documentation of the genomic architecture of the current composite breeds and their evolution from the ancestral populations. It is, therefore, worthwhile for inventory purposes and to guide future breed development initiatives to investigate the breed relationships and differentiations and the genomic regions targeted by selection through breed development of the SAM and Merino-derived breeds. The study by Ciani et al. (2015) suggested that intensive gene flow, founder effects, and geographic isolation are the main factors that determined the genetic makeup of current Merino and Merino-derived breeds.

The Ovine SNP50 genotyping array and other similarly designed bead chips provide unprecedented power to scan the genomes of livestock and investigate footprints of selection and their impact on the genetic potential of breeds to meet designed production goals. With the advent of genome-wide SNP genotyping, different statistical methods have been developed to interrogate genomes for signatures of selection and the associated effects on phenotypes. Signatures of selection in a genome are usually associated with either high-frequency-derived alleles or highly differentiated allele frequencies between populations or long-range haplotypes with strong linkage disequilibrium (LD) (Grossman et al., 2010). Statistical methods such as the within-population-integrated haplotype homozygosity score (*iHS*) (Voight et al., 2006) and the between-population *Rsb* and *XP-EHH* test (Tang et al., 2007) have been developed to screen for high LD and long-range haplotype and infer on signatures of selection. The hapFLK package detects selection signatures based on differences in haplotype frequencies between all the populations (Fariello et al., 2014). A number of studies have used *iHS*, *Rsb*, and other similar methods in the investigation of selection sweeps in sheep (Paim et al., 2018; Alvarez et al., 2020), cattle (Chen et al., 2016; Tijani et al., 2019), and other livestock species.

A number of previous studies have contributed towards the genomic characterisation of Merino and Merino-derived breeds. The study by Liu et al. (2022) used whole-genome sequences of 10 South African Mutton Merino (SAMM) sheep together with 39 Australian Merino and Chinese Merino (wool-type Merino) sheep to identify selection signatures using the iHS and *XP-EHH* methods. On the other hand, the study by Megdiche et al. (2019) used a multi-cohort approach, comparing wool-type Merino-derived breeds with non-Merino-derived breeds raised in the same geographical regions using F_ST_ outlier methods, local ancestry approaches, and genome-wide patterns of distribution of runs of homozygosity (ROH) to infer on selection signatures. The South African Merino-derived breeds are a composite of Merino breeds and non-Merino-type breeds of predominantly fat-tailed Damara, Nguni, and Ronderib Afrikaner and other local breeds. In order to complement and add more information on Merino and Merino-derived breeds, our study included the presumed non-Merino and presumed ancestors of the Merino-derived breeds in the analysis. The addition of the non-Merino-type breeds, which is unique to this study, allowed for a comprehensive analysis and interpretation of the divergence of the Merino-derived breeds from their presumed ancestors. Therefore, this study aimed to investigate the population genetic structure, breed similarities, and divergence of Merino-derived sheep breeds in South Africa. As such, this study first investigated population structure and admixture levels by referencing the composite breeds of AFR, DM, and Meatmaster against their presumed ancestral breeds of Merino, South African Merino (SAM), South African Mutton Merino (SAMM), Ronderib Afrikaner (RDA), Damara, and Nguni. Based on this analysis, the study went on to investigate regions habouring selection sweeps within the composite breeds and between each composite breed and its presumed ancestors. In addition to the iHS (Voight et al., 2006) and *Rsb* and *XP-EHH* (Tang et al., 2007) methods used by Liu et al. (2022), which investigated signatures of selection within populations and between pairs of populations, respectively, this study used the hapFLK (Fariello et al., 2014) method for a global analysis of signatures of selection in a data set consisting of both Merino-type and the non-Merino presumed ancestors. SNP genotypes for this analysis were limited to those generated in previous studies (Nxumalo et al., 2018; Dzomba et al., 2020) and the International Sheep Genomics Consortium HapMap data (http://www.sheephapmap.org). The study hypothesised that crossbreeding, followed by intensive selection towards breed-specific production goals, resulted in genomic divergence between the South African Merino and Merino-derived breeds.

## Materials and methods

### Animal genotypes

The study used Ovine SNP50K genotype data from a total of 279 animals obtained from five different sheep populations consisting of Merino (*n* = 46), SAMM (*n* = 8), SAM (*n* = 10), DM (*n* = 50), AFR (*n* = 52) and Meatmaster (*n* = 47), Nguni = (*n*=29), RDA (*n* =17) and Damara (*n* =20). The AFR, DM, Meatmaster, SAM, and SAMM genotypes were obtained from samples kept in a biobank at Grootfontein College of Agriculture, South Africa, together with the Nguni sheep that were sampled from the KwaZulu-Natal region of South Africa, and the SNP genotype data were generated and reported in a previous study by Dzomba et al. (2020). The Damara sheep genotypes were provided from a separate study (Nxumalo et al., 2018), while the RDA and Merino genotypes were extracted from the ISGC (http://www.sheephapmap.org). The AFR, DM, and Meatmaster are the Merino-derived composite breeds, and their presumed ancestral breeds based on literature (Synman, 2014a-d) are presented in Table 1.

**TABLE 1:** South African Merino-derived breeds and their ancestral breeds.

Composite breed	SAMM (8)	SAM (10)	Merino (46)	RDA (17)	Damara (20)	Nguni (29)
Afrino (52)	X	X	X	X		
Dohne Merino (50)	X	X	X			
Meatmaster (47)	X	X			X	X

### Genotype data quality control

In this study, the Illumina OvineSNP 50K BeadChip genotypes (as reported by Nxumalo et al. (2018); Dzomba et al. (2020); and ISGC, http://www.sheephapmap.org) were subjected to quality control using PLINK v1.07 (Purcell et al., 2007) and Golden Helix SVS v8.1 (Golden Helix, Inc., Bozeman, MT, www.goldenhelix.com) to ensure all SNPs had less than 5% missing genotypes, a call rate more than 95%, a minor allele frequency (MAF) less than 5%, and in Hardy–Weinberg equilibrium (*p* < 0.0001). Additional quality control measures ensured that individual animals had an IBD <0.025. High levels of pairwise linkage disequilibrium (LD) between SNPs may affect both the performance and efficiency of genomic prediction models. Therefore, in order to minimise bias for SNPs in strong linkage disequilibrium (*r* ^*2*^ > 0.2), the second SNP was removed, leaving a dataset with SNP pairs whose *r* ^*2*^ < 0.2 (Purcell et al., 2007).

### Determination of within-breed genetic diversity

To determine the expected (*H*_*E*_) and observed heterozygosity (*Ho*) values, PLINK v1.07 (Purcell et al., 2007) was used by running the command *“--hardy”* on the data for each breed. Inbreeding coefficients were calculated as the difference between expected (*H*_*E*_) and observed heterozygosity (*Ho*) values divided by the expected heterozygosity (*H*_*E*_) values also in PLINK v1.07. The mean *H*_*E*_ and *Ho* values per breed were calculated using the PROC MEANS procedure in SAS (2013).

### Analysis of molecular variation

Analysis of molecular variance (AMOVA) was used to determine the genetic variance within populations (*F*_*IS*_), among populations within groups (*F*_*SC*_), and among groups (*F*_*CT*_) using Arlequin v3.5 (Excoffer and Lischer, 2009). The populations were categorised into (i) all nine breeds; (ii) composite breeds of the AFR, DM, and Meatmaster; (iii) the presumed ancestral populations of the Nguni, Damara, RDA, SAMM, SAM, and Merino; and (iv) each composite breed and its presumed ancestors, which included (a) the AFR and Ronderib Afrikaner, Merino, and SAMM; (b) the Meatmaster, Damara, Nguni, SAMM, and SAM; and (c) the DM, SAM, SAMM, and Merino.

### Analysis of population structure

Principal component analysis (PCA) was carried out using Golden Helix SVS v8.1 (Golden Helix, Inc., Bozeman, MT, www.goldenhelix.com). The eigen values and eigen vectors for the principal components were estimated using Golden Helix SVS v8.1 (Golden Helix, Inc., Bozeman, MT, www.goldenhelix.com).

### Inference of local genomic ancestry (PCAdmix)

PCAdmix v1.0 (Brisbin et al., 2012) was used to infer local genomic ancestry in the composite breeds. The program utilises haplotypes from ancestral representatives to infer the ancestry of focal individuals. In this study, the SAMM, SAM, Merino, and RDA were treated as the ancestral representatives of the AFR sheep, the SAMM, SAM, and Merino breeds were treated as the ancestral representatives of the DM, and the SAMM, SAM, Damara, and Nguni were treated as the ancestral representatives of the Meatmaster. The software algorithms perform the inference chromosome-wide through PCA, *via* short windows along each chromosome. Using a hidden Markov model, PCAdmix then returns the posterior probability (PP) of ancestry from each reference population for each haploid individual in each window. PCAdmix requires phased genotypes, which were obtained using fastPHASE v1.2 (Scheet and Stephens, 2006) with default parameters.

### Analysis of selective sweeps and differentiating genomic regions

Selection signature analysis was used to assess genome-wide signatures of selection in the composite Merino-derived sheep of South Africa. The hapFLK package v1.2 was used to detect selection signatures based on differences in haplotype frequencies between all the Merino-derived breeds included in this study (Fariello et al., 2014). The number of haplotype clusters (*K*) was calculated using the imputeqc R package and accompanied scripts (Khvorykh and Khrunin, 2020). Using the number of haplotype clusters, the hapFLK values and the kinship matrix were calculated in the fastPHASE model (-*K* 40). Since the implementation of this approach required the construction of a neighbour-joining (NJ) tree from using a kinship matrix, Reynolds genetic distances were converted into the kinship matrix using an R script supplied with the package. In the construction of the NJ tree, Afrino was used as the outgroup population. The hapFLK statistic was computed as the average value across 40 expectation maximization (EM) runs to fit the LD model. The *p*-values were obtained by running a Python script “Scaling_chi2_hapFLK.py” available at forge-dga.jouy.inra.fr/documents/588, which fits a chi-squared distribution to the empirical distribution. The Manhattan plot was rendered in R using the qqman package (Turner, 2014) (settings for the suggestive line *q* = 0.03 and genome-wide line *q* = 0.01) to indicate selection.

The selection signature analysis also included complementary extended haplotype-based statistics (EHH-based statistics). The integrated haplotype score (iHS) approach compares EHH between alleles at an SNP within a population (Voight et al., 2006), while the extended haplotype-based homozygosity (*Rsb*) and cross population extend haplotype homozygosity (*XP-EHH*) approaches compare EHH patterns of the same allele between two populations (Tang et al., 2007). The ancestral alleles required for the computation were inferred as the most common alleles in the entire dataset, as previously described (Bahbahani et al., 2015). Haplotypes were phased using Beagle (Browning and Browning, 2007) and used to calculate *iHS* scores for each SNP/haplotype within a breed/population and *Rsb* and *XP-EHH* scores for each SNP/haplotype between breeds/populations. Haplotype frequencies were calculated using sliding windows of 20 SNPs that overlapped by five SNPs. For each locus, the *iHS*, *XP-EHH*, and *Rsb* scores were computed using the REHH package (Gautier and Vitalis, 2012) in R. For the analysis of within-population (*iHS*) and between-population differences (*Rsb* and *XP-EHH*), a score > 3 (i.e., a –log_10_ 3 score corresponding to a two-sided *p*-value < 0.001) was used to infer the candidate genomic regions under selection.

### Mapping the region of differentiation to find genes

Under selection SNPs *(p<0.001*) were mapped for genes using the Ensembl genome browser and NCBI (NCBI; www.ncbi.nlm.nih.gov). Ensembl Ovine (*Ovis aries*) genome build OAR3 was implemented in Golden Helix SVS v8 (Golden Helix, Inc., Bozeman, MT, www.goldenhelix.com)*.* Candidate genes were considered if their boundaries fell within 75 kb up or downstream of the selection sweep region defined. The associated genomic regions were also annotated using the Sheep QTL database (www.animalgenome.org/cgi-bin/QTLdb/OA/summary).

## Results

### Genetic diversity

All 277 animals proceeded for further analysis following quality control. The number of SNPs retained for analysis ranged from 36,976 in the Ronderib Afrikaner to 37,671 in the AFR (Table 1). Highest genetic diversity values were observed in the DM with *H*_*o*_ = 0.39 ± 0.01 followed by the Meatmaster and SAM with *H*_*o*_ = 0.37 ± 0.03. Lowest diversity was observed in the Nguni with *H*_*o*_ = 0.28 ± 0.02. Inbreeding estimates ranged from 0.00 ± 0.02 in the DM to 0.27 ± 0.05 in the Nguni (Table 2).

**TABLE 2:** Expected and observed heterozygosity in five sheep breeds of South Africa.

Breed	Number of animals	Number of SNPs	*H*_*E*_ *±SD*	*H*_*O*_ *±SD*	F_IS_
Afrino	52	37,671	0.39 ± 0.00	0.36 ± 0.01	0.06 ± 0.02
Meatmaster	47	36,586	0.39 ± 0.00	0.37 ± 0.03	0.05 ± 0.07
Merino	46	37,686	0.39 ± 0.00	0.35 ± 0.01	0.07 ± 0.03
SA Merino	10	37,452	0.39 ± 0.00	0.37 ± 0.03	0.04 ± 0.08
SA Mutton Merino	8	37,614	0.39 ± 0.00	0.34 ± 0.02	0.12 ± 0.04
Dohne Merino	50	37,638	0.39 ± 0.00	0.39 ± 0.01	0.00 ± 0.02
Damara	20	37,626	0.39 ± 0.00	0.31 ± 0.02	0.19 ± 0.04
Ronderib Afrikaner	17	36,976	0.39 ± 0.00	0.33 ± 0.03	0.14 ± 0.07
Nguni	29	37,634	0.39 ± 0.00	0.28 ± 0.02	0.27 ± 0.05
All breeds	279	37,381	0.39 ± 0.00	0.35 ± 0.03	0.08 ± 0.09

### Analyses of molecular variation in pure and developed breeds

Table 3 illustrates the partitioning of variation within breeds, among breeds, and among breeds within the categories of (i) ancestral breeds, (ii) composite breeds, and (iii) each composite breed and its presumed ancestors. Within-population variation was found to be 90% in the composite breeds and 84% within the presumed ancestral breeds, whilst it was 83% within all breeds (Table 3). A high level of molecular variation was observed within populations in comparison to among populations and among individuals within populations. In the category of a breed and its presumed ancestors, the highest within-population variation was observed in the AFR and its presumed ancestors (92%), followed by the DM category (90%) and least in the Meatmaster category (77%). Among-breed variation within groups was highest in the Meatmaster (21%) and its presumed ancestors, followed by the group of ancestral breeds (15%), and least in the AFR and its presumed ancestors (6.4%). Within-breed variation was highest in the category consisting of all eight breeds (17%) and least in the category with ancestral breeds.

**TABLE 3:** Analysis of molecular variation.

Dataset	Variance component (%)		
	Among breeds (*F*_*CT*_)	Among breeds within groups (*F*_*SC*_)	Within breeds (*F*_*IS*_)
All eight breeds	10.62 (17.23%)	-	91.52 (82.77%)
Ancestral breeds	0.011 (1.06%)	0.149 (14.77%)	0.158 (84.17%)
Composite breeds	0.029 (2.97%)	0.071 (6.91%)	0.099 (90.11%)
Afrino and presumed ancestors	0.014 (1.42%)	0.065 (6.43%)	0.079 (92.15%)
Meatmaster and presumed ancestors	0.021 (2.12%)	0.218 (21.38%)	0.235 (76.50%)
Dohne Merino and presumed ancestors	0.048 (90.21)	0.052 (4.95%)	0.098 (90.21%)

### Analysis of population structure

**FIGURE 1:**
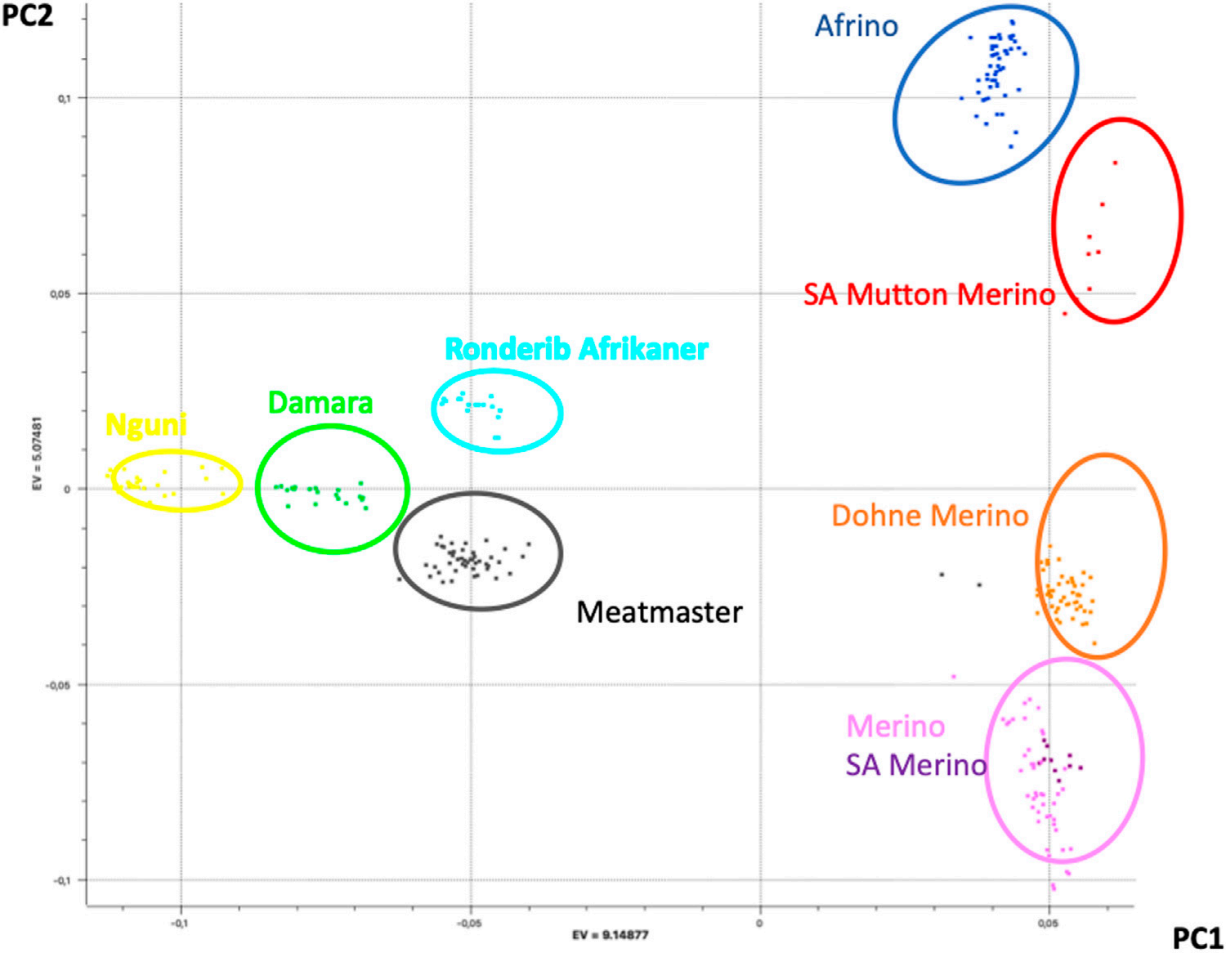
PCA-based clustering of Merino, Merino-derived breeds, and non-Merino presumed ancestors.

The PCA results that explained the population structure (i.e., PC1 and PC2) explained 43% of the total variance (Figure 1). While PC1 (28% of the variation) separated the Merino, SAM, SAMM, DM, and AFR from the Meatmaster, Damara, Nguni, and Ronderib Afrikaner, PC2 (15% of the variation) separated the Damara, Meatmaster, Merino, SAM, and DM from the SAMM, Ronderib Afrikaner, and AFR. In PC1, the AFR was on the same axis as its Merino ancestors, but separated from the RDA. The DM clustered with the two Merinos, while the Meatmaster, on the other hand, clustered in the same axis with its Nguni and Damara presumed ancestors separated from the Merinos.

### PCAdmix-based analysis of co-ancestry

**FIGURE 2:**
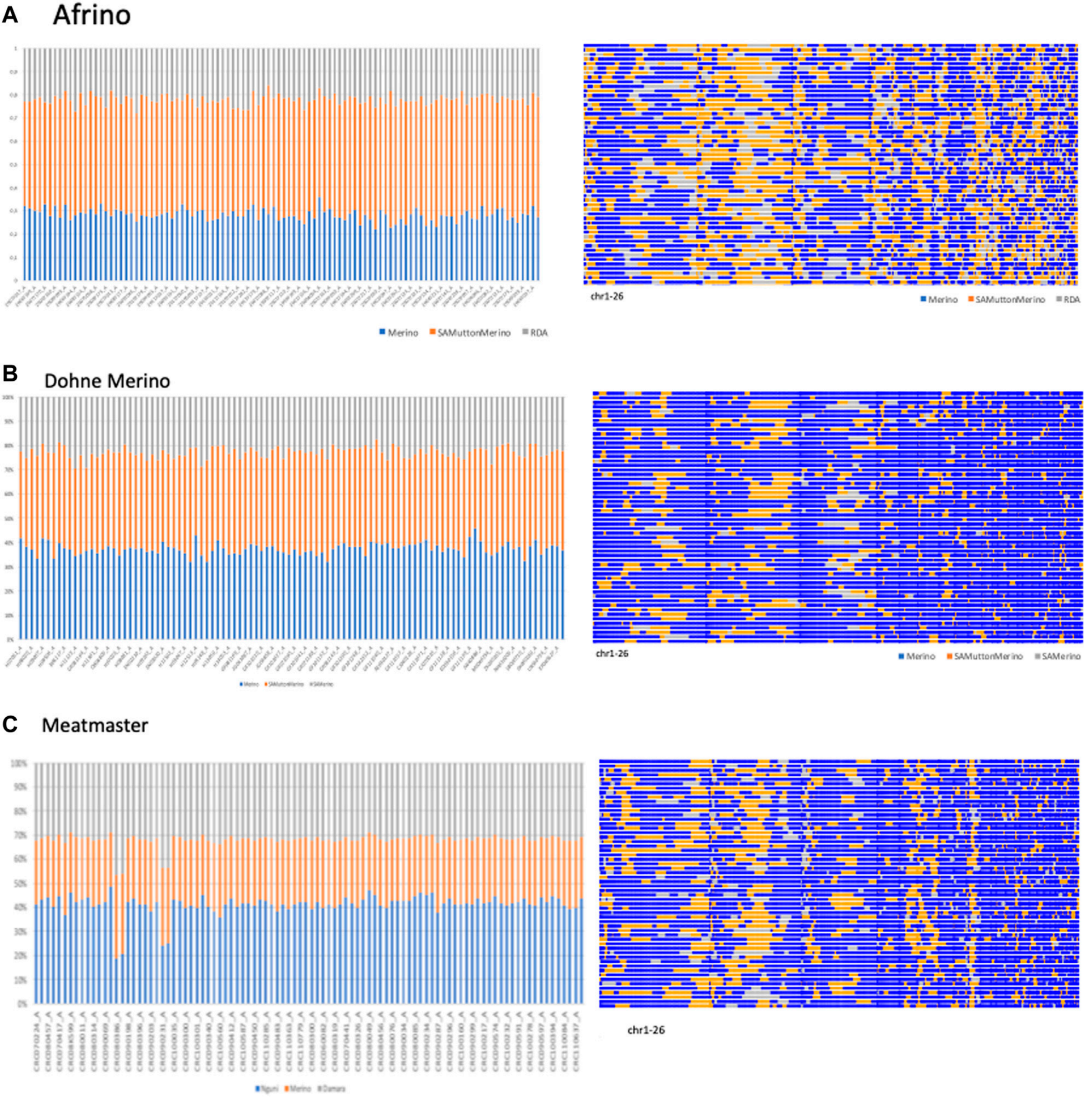
PCAmix of Merino-derived breeds [Afrino, **(A)**; Dohne Merino, **(B)**; and Meatmaster, **(C)**] and their presumed ancestors.

The PCAdmix results are illustrated in Figure 2. Using the PCAdmix algorithm, the genome of each composite Merino-derived breed was partitioned into segments of inferred ancestry at a resolution of chromosomal level. The PCAdmix of the AFR yielded tracts of ancestry consistent to predominantly SAMM (49.7%, 2.8 ± 0.03) followed by Merino (28.3%, 2.5 ± 0.03) and RDA (21.9%, 2.3 ± 0.02) (Figure 2A), consistent with a targeted ratio of 50:25:25 of the respective contributing breeds. The DM that was developed from crossing SAMM and the SAM ewes was predominantly Merino (37.4%, 2.4 ± 0.03) and SAMM (39.8%, 2.6 ± 0.03) and less of the SAM (22.8%, 2.4 ± 0.02) (Figure 2B). The Meatmaster, a composite of many breeds (Peters et al., 2010), was largely Nguni (41.2%, 2.4 ± 0.04) and Damara (32.0%, 2.9 ± 0.03) and less of the Merino (26.8%, 1.8 ± 0.02) breeds (Figure 2C).

### Signatures of selection

**FIGURE 3:**
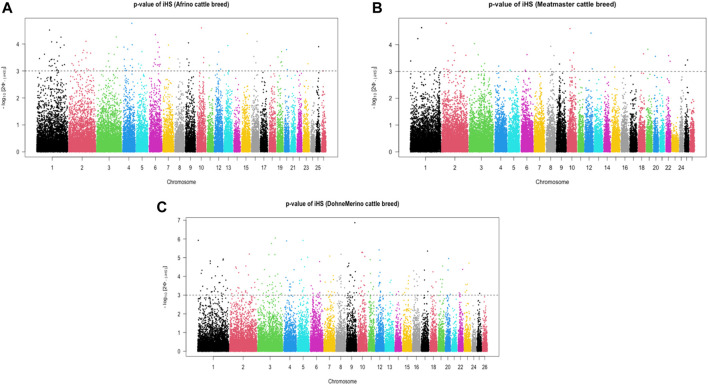
*iHS* scores of **(A)** Afrino, **(B)** Meatmaster, and **(C)** Dohne Merino.

The analysis of within-population iHS >3.0 identified 21 selection sweep regions distributed across 14 chromosomes (OAR 1, 2, 3, 4, 5, 6, 8, 9, 10, 12, 13, 15, 16, and 19) in the DM sheep (Figure 3; Table 4; Supplementary Table S1). Using this method, fewer selection sweep regions in the Afrino and Meatmaster were identified. Only nine selection sweep regions distributed across eight chromosomes (OAR 1, 2, 3, 4, 6, 8, 13, and 19) were identified in the Afrino, while only four selection sweep regions across four chromosomes (OAR 1, 2, 3, and 9) were identified in the Meatmaster (Figure 3).

**TABLE 4:** *iHS*-based selection sweep regions and associated genes in Afrino, Dohne Merino, and Meatmaster sheep breeds and Rsb, XP-EHH and hapFLK-based selection sweep regions for Afrino, Dohne Merino, Meatmaster sheep breeds and their presumed ancestors.

	Start	Stop	Method	QTL trait (QTLID)*	Candidate gene
1	119.6	138.1	iHSDM; iHSAfrino	Muscle weight in carcass (14276), carcass fat percentage (14277, 14251, and 14277), lean meat yield percentage (14278), and bone weight in carcass (14275)	SLC5A3; MRPS6; IFNGR2; IFNAR1; IL10RB, PAXBP1; SYNJ1; MIS1BA; HUNK; SCAF4, S15; CHODL; BTG3; CXADR
1	168.6	201.5	iHSDM; iHSAfrino; Rsb_DM/SAMM; Rsb_Afrino/SAMM	Lean meat yield percentage (14278 and 14252), carcass fat percentage (14277 and 14251), reproductive seasonality (16602), and muscle weight in carcass (14320, 14270, and 14250)	*ALCAM*; *CBLB*; *HEG1*; *ZNF148*; *LRCH3*; *SLC49A4*; *SLC51A*; *AP; FGF12*; *GMNC*; *PLAAT1; IL1RAP; TPRG1; ATP13A4*
1	212.1	244.1	iHSMM; iHSDM; Rsb_DM/Merino; XP-EHH_DM/Merino; Rsb_Afrino/Merino; Rsb_Afrino/RDA; XP-EHH_DM/Merino; XP-EHH_Afrino/Merino; XP-EHH_MM/Merino	Carcass fat percentage (14277), lean meat yield percentage (14252), and reproductive seasonality (16603)	*SPATA16; ECT2; GHSR; SLC2A2; SLC7A14; SHOX2; VEPH1; PTX3; CCNL1*
1	263.4	285.0	Rsb_DM/Merino; XP-EHH_DM/Merino; Rsb_MM/Nguni; XP-EHH_MM/Nguni; Rsb_MM/Merino; XP-EHH_MM/Merino; XP-EHH_Afrino/Merino hapFLK	Carcass fat percentage (14277), reproductive seasonality (16603), average daily gain (13948, 13955, and 13964) , and *Trichostrongylus colubriformis* FEC (12884)	*IGSF10; P2RY12; P2RY14; WWTR1; RNF13; GPR171; DIPK2A; SLC9A9; CH5T2; SETD4; CBR3; CAPN7*
2	6.2	26.0	iHSMM; iHSDM; Rsb_Afrino/RDA; XP-EHH_DM/Merino; XP-EHH_Afrino/Merino; XP-EHH_Afrino/SAMM; XP-EHH_MM/Merino	Body weight (57659, 14171, and 14280), average daily gain (57776), hot carcass weight (14279), hindquarter weight (14161), subcutaneous fat thickness (13722), loin fat weight (13732); and meat color (14163, 14167, and 14165)	*TMC1; ALDH1A1; FAM189A2; HPF1; CLCN3; NEK1*
2	62.0	109.5	iHSMM; iHSDM; XP-EHH_Afrino/RDA	No hit	
2	130.9	168.6	iHSDM; iHSAfrino XP-EHH_DM/Merino; XP-EHH_DM/SAMerino; XP-EHH_Afrino/Merino	Hot carcass weight (14279), meat color (14163, 14165, 14169, 14164, and 14168), longissimus muscle area/width/weight (13728, 13726, 13729), loin fat thickness (13730), and subcutaneous fat weight (13731 and 13738)	*LRP1B*
3	15.4	17.2	Rsb_DM/SAMerino; XP-EHH_DM/SAMerino	*Trichostrongylus colubriformis* FEC (14155), and body weight (56 weeks) (13927)	*SOX11; RSAD2; RNF144A*
3	45.9	74.3	Rsb_Afrino/RDA; Rsb_DM/Merino; XP-EHH_DM/Merino; XP-EHH_MM/Merino; XP-EHH_Afrino/Merino	Internal fat amount (14281) and body weight (56 weeks) (13927)	*LRRTM4; XPO1; FAM161A; NRXN1; U6*
3	114.5	153.3	iHSAfrino; iHSDM; iHSMM	Internal fat amount (14281 and 14255), *Trichostrongylus colubriformis* FEC (12885) , meat-conjugated linoleic acid content (17220), and body weight at birth (17230)	*SYT1; PPP1R12A; ZNF641; PFKM; SLC48A1; COL2A1; HDAC7; SLC38A4; SLS38A2; SLC38A1; ARID2; YEATS4; LYZ; KCNMB4; SLC35E3; RAP1B; CCT2; MYRFL; IL22; IL26*
4	16.1	63.5	iHSAfrino; iHSDM	Body weight (17232), *Haemonchus contortus* FEC (19803)	*ICA1; NXPH1; AVL9; NT5C3A; FKBP9; PDE1C*
5	23.8	85.5	iHSDM; Rsb_MM/Merino; hapFLK<	Body weight (birth) (12934) and body weight (20 weeks) (193069)	*SLC12A2; MEGF10; PHAX; TEX43; PRSS57; PLPPR3; RNF126; STK11; REDX01*
6	26.1	38.5	iHSDM; Rsb_Afrino/RDA; XP-EHH_Afrino/RDA; XP-EHH_Afrino/Merino; XP-EHH_DM/Merino; XP-EHH_MM/Damara	Fat weight in carcass (95819 and 95820), total fat area (95836, 95837, 95826, 95827, and 95828), fat density (95850), and body weight (14261, 14284, 193062, 193068, and 193063)	*STPG2, CCSER1; MMRNA1; SNCA; FAM13A; ABCG2; SPP1; LCORl*
6	41.3	64.4	iHSAfrino; Rsb_Afrino/RDA; XP-EHH_Afrino/RDA XP-EHH_DM/Merino; XP-EHH_MM/Nguni	Fat weight in carcass (95822 and 95823), total fat area (95838, 95839, and 95841), fat density (95840 and 95842), and muscle density (95864)	*GBA3; PPARGC1A; DHX15; SOD3; LG12*
6	93.5	102.3	iHSAfrino; iHSDM	Body weight (slaughter) (14284), *Trichostrongylus colubriformis* FEC (12887), reproductive seasonality (195222), and lean meat yield percentage (14286)	*GUF1; YIPF7; GNPDA2; GABRA4; ANTXR2; FGF5; GK2; FGFS; SLC10A6; PTPN13; MAPK10*
7	37.2	93.0	XP-EHH_MM/Damara; XP-EHH_DM/Merino	No hit	
8	47.4	51.8	iHSAfrino; iHSDM; XP-EHH_DM/Merino	*Trichostrongylus* adult and larva count (12899 and 12900) and internal fat amount (14288)	*LYRM2; BACH2; GJA10; GABRR1; RARS2; SLC35A; SPACA1*
9	31.8	40.7	iHSMM; iHSDM; XP-EHH_MM/Nguni	Hot carcass weight (14290) and longissimus muscle area (14323)	*SPIDR; PRKDC; MCM4, RGS20; SOX17; TGS1; TCEA1;*
10	14.5	32.5	Rsb_MM/Nguni; XP-EHH_MM/Nguni; Rsb_DM/Merino; XP-EHH_DM/Merino; Rsb_MM/Merino; XP-EHH_MM/Merino; XP-EHH_DM/SAMerino; XP-EHH_Afrino/Merino	Tail fat deposition (127009), milk fat yield (QTL:169583), ear size (QTL:159964), total lambs born (QTL:130451), horn circumference (QTL:161397), and horn type (QTL:161480)	*RXFP2; RXFP2; FRY; BSGLCT*
10	34.5	59.4	iHSDM; Rsb_Afrino/RDA; Rsb_MM/Nguni; Rsb_DM/SAMM; Rsb_Afrino/SAMM; XP-EHH_DM/SAMM XP-EHH_MM/SAMM; P-EHH_Afrino/SAMM XP-EHH_Afrino/Merino; XP-EHH_MM/SAMerino; XP-EHH_Afrino/RDA; XP-EHH_MM/Damara XP-EHH_MM/Nguni	Fat weight in carcass (14292), carcass bone percentage (14293), carcass fat percentage (14294), and lean meat yield percentage (14295)	*SPRY2; SLITRK4; SLITRK5; SLC16A9; BORA; PIBF1; MZT1*
10	59.6	72.1	iHSDM; Rsb_Afrino/RDA; XP-EHH_Afrino/RDA; Rsb_MM/Nguni; XP-EHH_MM/Nguni; XP-EHH_Afrino/Merino XP-EHH_Afrino/SAMM XP-EHH_MM/Damara XP-EHH_MM/SAMM; hapFLK	Body weight (57656), fat weight in carcass (14292), carcass bone percentage (14293), carcass fat percentage (14294), and lean meat yield percentage (14295)	*GPC5; SLITRK5; SLC16A9; GPR180; GPC5; TGDS; DCT; CTNNA3; GCD; TGDS; RUFY2; SLC25A16; COX20P*
10	78.6	81.7	Rsb_DM/Merino XP-EHH_DM/Merino Rsb_MM/Merino XP-EHH_MM/Merino XP-EHH_Afrino/Merino	Fat weight in carcass (14264), carcass fat percentage (14265), and lean meat yield percentage (14266)	*POGLUT2; SLC10A2*
11	21.5	38.2	Rsb_Afrino/SAMM; XP-EHH_Afrino/Merino; Rsb_DM/Merino; XP-EHH_DM/Merino; Rsb_DM/SAMerino; Rsb_DM/SAMM; XP-EHH_DM/SAMM; Rsb_MM/Merino; XP-EHH_MM/Merino	Internal fat amount (14298), body weight (14297), average daily gain (13945 and 13966), and hot carcass weight (14296)	*MAP2K4; DNAH9; MYH3; MYH4; MYH8; NCOR1; ZNF624; SLC47A2; SLC5A12; MAPK7; FSHB; SOX15; SAT2; SNORA62; SOX; SLC13A5; TEKT1*
12	28.3	78.1	iHSDM; XP-EHH_Afrino/Merino; XP-EHH_DM/Merino	Body weight (yearling) (213860)	*VAMP4; FMO4; TNFSF18; ACOT7; NOL9; HES3; RNF207*
13	36.5	53.7	iHSAfrino; iHSDM; Rsb_Afrino/RDA; XP-EHH_ Afrino/RDA; XP-EHH_Afrino/Merino; hapFLK	Muscle weight in carcass (14301); tail fat deposition (127011)	BFSP1; RRBP1; DSTN; SNX5; KAT14; OVOL2; RASSF2; SLC23A2; BMP2
14	27	30.9	Rsb_MM/Nguni; XP-EHH_MM/Nguni	Dressing percentage (14304 and 14270), bone weight in carcass (14302), and fat weight in carcass (14269)	*CDH8*
16	33.5	45.8	iHSDM; XP-EHH_DM/SAMerino	Subcutaneous fat thickness/area (14309/14308), body weight (slaughter) (14306), and Dressing percentage (14305)	
19	25.8	27.3	iHSDM XP-EHH_DM/SAMerino	Average daily gain (193081)	
19	41.2	48.8	iHSAfrino; iHS|DM; Rsb_MM/Nguni; XP-EHH_MM/Nguni	Entropion (193397, 193378, and 193385)	*FAM3D; FAM107A; C3or167; ACOX2; WNT5A; IL17RD; IL17RB; ACTR8; DCP1A; SELENOK; CACNA1D; NEK4; SPCS1*

* The associated genomic regions were annotated using the Sheep QTL database (www.animalgenome.org/cgi-bin/QTLdb/OA/summary).

**FIGURE 4:**
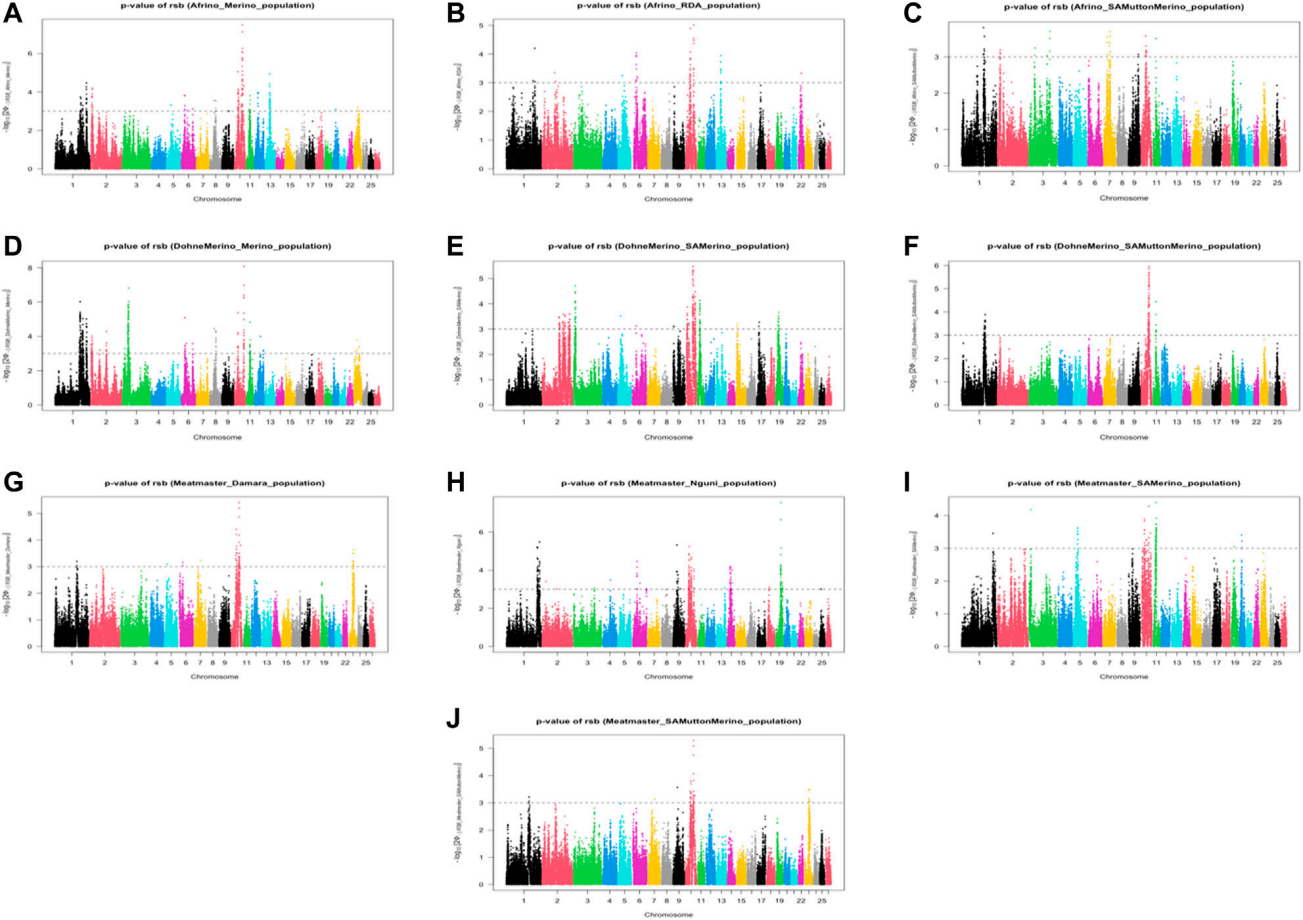
Genome-wide XP-EHH and Rsb scores of Merino-derived breeds and against their presumedancestors. Afrino and Merino **(A)**, Afrino and RDA **(B)**, Afrino and SAMuttonMerino **(C)**, Dohne Merino and Merino **(D)**, Dohne Merino and SAMerino **(E)**, Dohne Merino and SAMuttonMerino **(F)**, Meatmaster and Damara **(G)**, Meatmaster and Nguni **(H)**, Meatmaster and SAMerino **(I)** and Meatmaster and SAMuttonMerino **(J)**.

**FIGURE 5:**
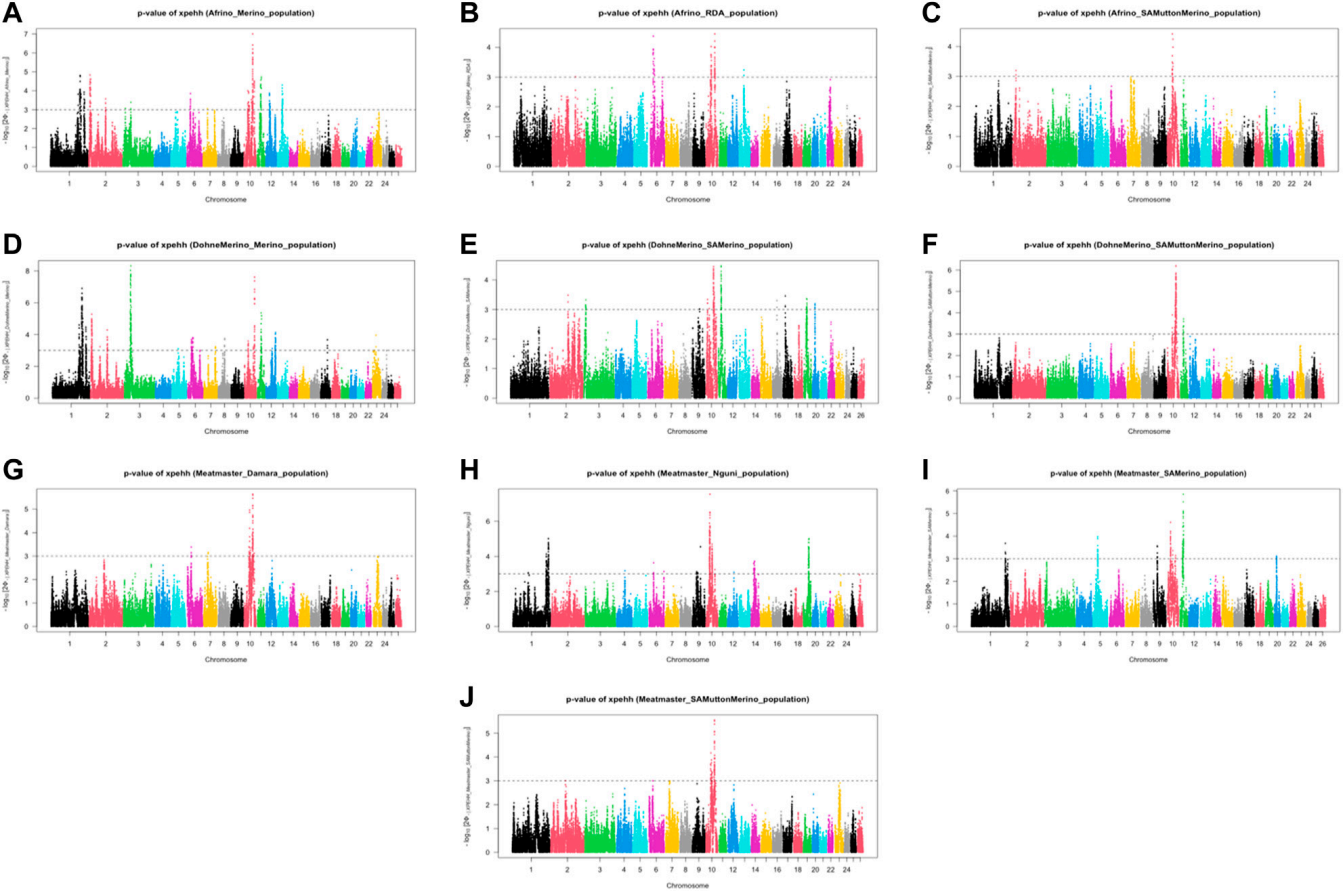
XP-EHH scores of Merino-derived breeds against presumed ancestors. Genome-wide XPGenome-wide XP-EHH scores of Merino-derived breeds and against ancestors. Afrino and Merino **(A)**, Afrino and RDA **(B)**, Afrino and **(C)**, Dohne Merino and Merino **(D)**, Dohne Merino and SAMerino Dohne Merino and SAMuttonMerino **(F)**, Meatmaster and Damara Meatmaster and Nguni **(H)**, Meatmaster and SAMerino **(I)** and Meatmaster and SAMuttonMerino **(J)**.

The analysis between the composite Merino-derived breed and each of its presumed ancestors (Rsb and XP-EHH >3.0) identified 25 selection sweep regions distributed across 15 chromosomes (OAR 1, 2, 3, 4, 5, 6, 7, 8, 9, 10, 11, 12, 13, 16, and 19) (Figure 4; Table 4; Supplementary Table S2, 3). Of the 25 selection sweep regions, 18 were identified using the *iHS* (>3.0) method. hapFLK results between the composite Merino-derived breed and each of its presumed ancestors identified eight selection sweep regions distributed across five chromosomes (OAR 1, 5, 10, 13, and 25) (Figure 5; Table 4; Supplementary Table S4). All five of the selection sweep regions identified using hapFLK analyses were also identified using the Rsb and XP-EHH methods (>3.0).

**FIGURE 6:**
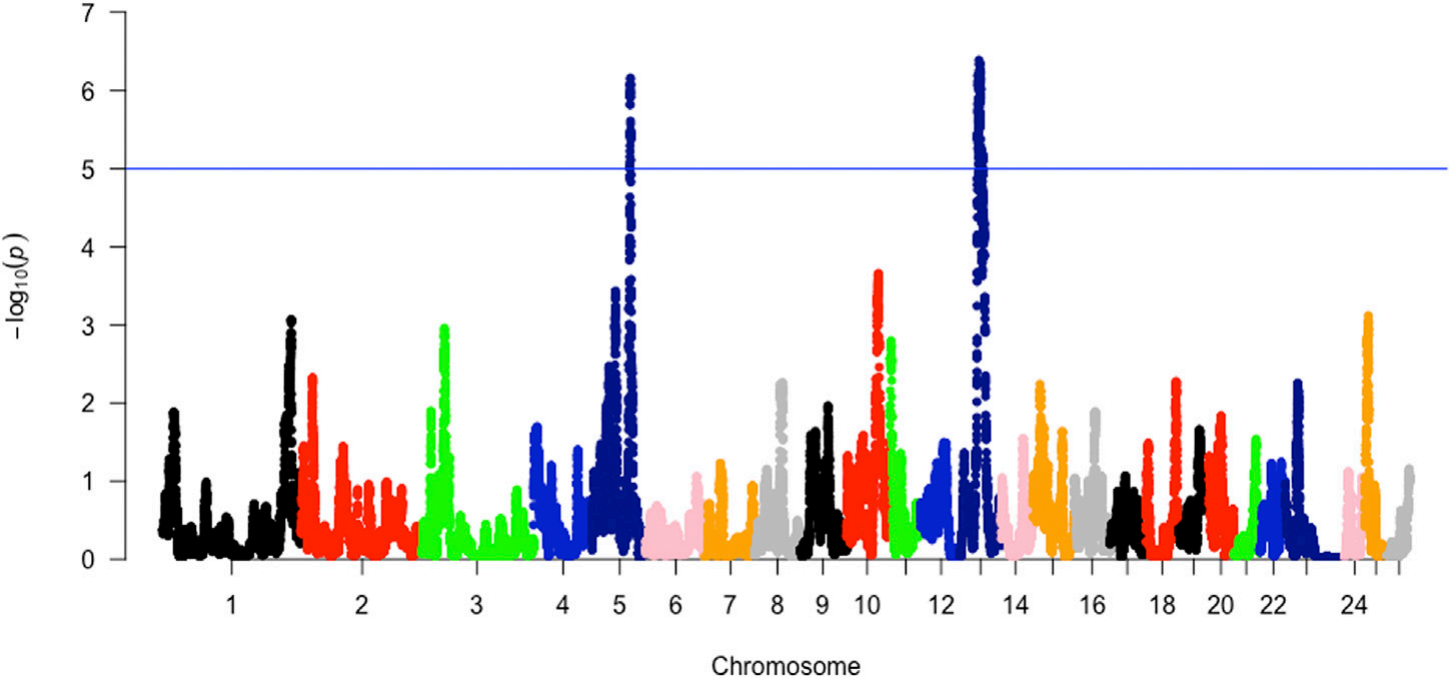
hapFLK scores of Merino-derived breeds against presumed ancestors.

Numerous genes and QTLs were identified in selection sweep regions. These included genes *CAPN7*, *IFNAR1*, *IL10RB*, *SLC2A2*, *SLC5A3*, *SLC7A14*, *CRYZL1*, *FGF12*, *GPR171 GHSR*, *and SPATA16* that were within the sweep on the OAR1 region and *HMGA*, *IL22*, and *IL26* on OAR3, while genes *ICA1* and *NXPH1* were observed within the OAR4 sweep; *FGF5* and *ANTXR2* on OAR6; *RXFP2*, *Y2*, *SLITRK4*, and *GPC* on OAR10; *MAP2K4*, *SPTSSB*, *PPMIL*, and *B3GALNT* on OAR11; *BMP2* on OAR13; and the *WNT5A* gene on OAR19. The associated genomic regions annotated using the Sheep QTL database (release 48) (www.animalgenome.org/cgi-bin/QTLdb/OA/summary) identified several QTLs associated with important health and production traits. This included QTLs associated with reproductive traits (e.g., reproductive seasonality, QTLID:16602, QTLID:16603, and QTLID:195222 and total lambs born, QTL:130451); skeletal morphology and body size (e.g., carcass bone percentage, QTLID:14293), body weight at birth, QTLID:12934; body weight at 20 weeks, QTLID:193069; muscle density, QTLID:95864; carcass fat percentage, QTLID:14277; ear size, QTL:159964); milk yield and quality traits (e.g., milk fat yield, QTL:169583); horn size and type traits (e.g., horn circumference, QTL:161397 and horn type, QTL:161480), and immune response (e.g., *Trichostrongylus colubriformis* FEC, QTLID:12884 and QTLID:4155; *Haemonchus contortus* FEC, QTLID:19803; *Trichostrongylus* adult and larva count, QTLID:12899 and QTLID:12900).

## Discussion

In conducting this study, we made use of available sheep genotypes from previous projects to make inferences on genetic diversity, breed relations, and divergence amongst the Merino-derived breeds and their presumed ancestors. Our data were drawn from previous studies reported for the SAMM, SAM, DM, Meatmaster, and AFR (Dzomba et al., 2020); for the Damara sheep (Nxumalo et al., 2018); and for the Ronderib Afrikaner and Merino sheep (Kijas et al., 2012). These Merino and Merino-derived sheep breeds dominate the South African sheep industry, contributing to mutton, wool, and other sheep by-products. Merino sheep originate from Spain and are primarily useful and highly prized for their wool. In South Africa, their use in livestock farming can be traced to the late 18th century when few founder ewes and rams were donated by the Dutch government for experimental purposes to the Cape Town government (merinosa.co.za/history/).

The current study focused to make inferences on genetic diversity. Observed and expected heterozygosity values together with the inbreeding coefficient were used to explain genetic diversity within the studied sheep breeds. The highest genetic diversity (*H*_*E*_) was observed in the DM followed by the Meatmaster, SAM, and AFR. The DM, AFR, and Meatmaster are the three Merino-derived composite breeds and are of high genetic diversity, similar to that reported in Spanish and Australian Merino breeds by Ciani et al. (2015). The Nguni, Damara, and Ronderib Afrikaner, which are the indigenous ancestral populations, are raised by few fragmented communities (Qwabe, 2011; Nxumalo et al., 2018; Selepe et al., 2018), which would explain the low levels of within-population genetic diversity. Although the founding populations of the Nguni, Damara, and Ronderib Afrikaner had low genetic diversity, crossbreeding them with Merino breeds to develop composite breeds resulted in increased diversity observed in the AFR and Meatmaster breeds, which can be attributed to the combination of two or more genetic pools. The results of this study clearly demonstrated that significant genetic variation is maintained in the composite Merino-derived sheep breeds.

To gain an insight into the genetic structure of breeds, analysis of molecular variation (AMOVA) was used to determine the portioning of variance within and between populations and within populations amongst groups. In panmictic populations, the variance is expected to come from within samples (Excoffer and Lischer, 2009). If the variance occurs among samples within the population or comes from among populations, this would be regarded as evidence of the existence of population structure (Excoffer and Lischer, 2009). As expected, within-population variation was high in the composite breeds. The AFR and DM were established from predominantly well-managed commercial breeds of SAMM, Merino, and SAM and, to a small extent, the Ronderib Afrikaner in the case of the AFR. These breeds have moderate-to-high genetic diversity (Table 2), which explains the high within-population genetic variation in this category. The Meatmaster, on the other hand, is based on the small and less diverse breeds of the Damara and the Nguni, which is reflected in the relatively lower (77%) within-population diversity in this group. Significant population substructure was, therefore, evident in the Meatmaster and its presumed ancestors’ category, with an among-breed diversity of 21%. These Merino-derived sheep breeds that exist as widely distributed admixed populations represent economically and historically important genetic resources (Ciani et al., 2015).

PCAdmix confirmed the presumed ancestry of the Merino-derived breeds of AFR, DM, and Meatmaster (Ciani et al., 2015). PC1 separated the Merino breeds from non-Merino breeds, with the exception of Meatmaster. While the Meatmaster was bred from fat-tailed sheep, there is intensive and directional selection against fat localisation and long tails in the breed (www.meatmasters.co.za). Coupled to this, part of the breed standards for the Meatmaster is that it should be 50% Damara (Synman, 2014c-d). These selection criteria explain its clustering with the Damara, Nguni, and Ronderib Afrikaner away from the Merinos and other Merino-derived breeds under PC1. In PC2 (15% of variation), the AFR clustered with the SAMM was separated from the DM, Merino, and SAM. According to the breed standards (http://www.afrino.org.za), 80% of the income from AFR is generated through meat production and 20% through wool production. This would be regarded as a biased selection objective towards growth and meat production traits, which explains why the AFR clustered with the SAMM. The Ronderib Afrikaner sheep are an improved form of the Namaqua Afrikaner sheep (Epstein, 1960), and together with the Damara and Nguni sheep are fat-tailed sheep (Peters et al., 2010), which could have formed the basis of their clustering together in PC1. Even though PCA is a statistical method commonly used in population genetics to identify structure in the distribution of genetic variation across populations, PCA projections are strongly influenced by uneven sampling (McVean, 2009), which might have been the case in this study. Despite the uneven sample sizes, the PCA clustering observed in this study resembles that observed in a prior study (Dzomba et al., 2020) that included more populations and sample sizes from which the investigated data set was sub-sampled, which validates the results. Similar clustering of Merino-type and non-Merino-type breeds was reported in other studies (Gifford-Gonzalez and Hanotte, 2011; Kijas et al., 2012). In spite of the importance of these breeds, there is limited information on the genomic influence of either Merino or other indigenous sheep on the different composite Merino-derived breeds. Results of this study that increase our knowledge regarding Merino-based breeds will, therefore, inform and guide future breed improvement programs and management and conservation efforts.

*iHS* analysis is used to infer recent and generally segregating selection sweeps (Voight et al., 2006) and has been used in humans (Liu et al., 2013) and a number of livestock studies for dairy cattle (Cheruiyot et al., 2018), pigs (Chen et al., 2016),etc. For AFR, *FGF12,* a candidate gene for hair follicle development (Lv et al., 2020) and reproductive traits (An et al., 2018), and *ICA1 and NXPH1*, associated with metabolic pathways (Akanno et al., 2018), were some of the key candidate genes identified. Although the AFR is predominantly selected or weighted for meat quality traits, the breed was established as a white-woolen breed for use as a terminal sire when crossed with Merino ewes (Synman, 2014) in response to the presence of kemp (coloured fibre) in crosses of Merino ewes with mutton breeds. The signature for hair follicle development might be a reflection of this selection. *ICA1* and *NXPH1*, on the other hand, are signatures of the intensive selection weight put on the AFR for meat traits (Synman, 2014).

The four selection sweep regions identified in the Meatmaster breed corresponded to *GPR171* on chromosome 1 which is associated with feed and metabolism (Ruiz-Larranaga et al., 2018). The *HMGA* gene on chromosome 3 was also observed as a selection signature in Sardinian ancestral black sheep (Kijas et al., 2012) and in Spanish breeds (Manunza et al., 2016). *HMGA2* is involved in skeletal morphology and body size (Kijas et al., 2012) and has been shown to be under selection in dogs with divergent stature (Jones et al., 2008; Akey et al., 2010). According to the breed society standards (www.meatmastersa.co.za/Breed-Standard.htm), Meatmaster sheep must be of average size and have a functional, efficient body conformation and well-placed legs with excellent walking ability. Such selection for body size and skeletal morphology could be the signature presented through the *HMGA2* gene, which, together with the *GPR171* gene associated with feed and metabolism, could ensure optimal performance for mutton production. Also, *IL22* and *IL26* on chromosome 3 are immune response genes that have been reported as under selection in some studies, including Fariello et al. (2014). Detection of immune response genes is especially expected in breeds raised in arid environments with harsh and compromised production systems (Mdladla et al., 2018).

Interestingly, more selection sweep regions were identified in the DM sheep relative to the AFR and Meatmaster. DM shared two sweeps with AFR, one on chromosome 1 in regions 119, 0–121, and 0 Mbs, which harbours *IFNAR1, IL10RB, SLC5A3*, and *CRYZL1* associated with anti-inflammatory and immune response (Uemoto et al., 2020) and reproductive traits such as implantation of the conceptus to the uterus (Zhang et al.*,* 2013), and one on chromosome 19 position in regions 46, 7–48, and 1 Mbs, which harbours the gene *WNT5A* (45 and 5 Mb), important in morphology, particularly the development of limbs and skeleton (Fariello et al., 2014) and reproductive traits (including mammary gland development) (Hao et al.*,* 2019). Other sweeps on chromosome 6 (93, 5–95, and 2 Mb) associated with the *FGF5* gene are reported as a signature of selection in worldwide sheep breeds by Kijas et al. (2012) and Fariello et al. (2014), and the *ANTXR2* gene is associated with adaptation to variation in climatic conditions (Lv et al., 2020). A selection sweep on chromosome 13 on regions 46, 5–48, and 1 Mb in the AFR and 49, 1–50, and 9 Mb in the DM was associated with the gene *BMP2*, which has been reported as a signature of selection by Kijas et al. (2012) and Fariello et al. (2014) and is strongly selected in both fat-tailed and thin-tailed sheep (Dong et al., 2020).

Rsb, XP-EHH, and hapFLK results presented selection sweeps between a composite Merino-derived breed and each of its presumed ancestors. Sweeps on OAR 1 yielded genes such as the *GHSR* important for growth and carcass traits in sheep (Bahrami et al., 2012); *SPATA16* is associated with environmental variables in goats (Mdladla et al., 2018) and male fertility in cattle (Wang et al., 2014); and the *SLC7A14* and *SLC2A2* are involved in nutrient transport and absorption (Wiedemar et al.*,* 2015). The selection sweep on chromosome 10 in regions 28, 6–30, and 3 Mb is associated with the *RXFP2* gene associated with polledness (Wang et al., 2014; Wiedemar and Drogenmuller, 2015). The region carried other genes such as the *FRY* gene, which is associated with lambing percentage, ear size, and coat phenotypes (Wei et al., 2015), and the *BSGLCT* gene, which is associated with wool traits. Zhang et al. (2013) suggested the role of *FRY* in sheep wool development. Overall, the Rsb, XP-EHH, and hapFLK analyses revealed the direction of selection when these breeds were selected, which focused on meat and wool production and robustness of breed through body confirmation, disease resistance, and adaptability to the harsh production conditions in South Africa (Kim et al., 2016; Molotsi et al., 2017).

Although fairly documented, there is limited information on the genomic influence of either Merino or other indigenous sheep on the different composite Merino-derived breeds. This study provided requisite information on the evolution of these composite breeds from their founding populations, which will inform and guide future breed improvement programs and management and conservation efforts.

## Data Availability

The raw data supporting the conclusions of this article will be made available by the authors, without undue reservation.
